# Association of demographics and psychological barriers with late presentation of HIV among men who have sex with men in Chengdu, China

**DOI:** 10.1038/s41598-025-33749-2

**Published:** 2025-12-26

**Authors:** Jing Zhang, Junguo Xin, Dezhi Guo, Lan Chen, Lingli He, Jing Tang, Yuhen Li, Jiachuan Wang, Xiaohong Yang

**Affiliations:** 1 Department of Public Health, Chengdu Medical College, Chengdu, 610000 China; 2 Department of Infectious Diseases, Nuclear Industry 416 Hospital (The 2nd Affiliated Hospital of Chengdu Medical College), Chengdu, 610000 China

**Keywords:** HIV infection, Men who have sex with men, Late presentation, Internalized homophobia, Self-stigma, Mental health, Diseases, Health care, Psychology, Psychology, Risk factors

## Abstract

In China, despite the improved availability of diagnostic services, men who have sex with men (MSM) still face high HIV risk and barriers to timely diagnosis due to internalized homophobia, self-stigma, and other potential factors. This study aimed to explore these factors and how they associated to late presentation (LP) of HIV among MSM in Chengdu, China. Characteristics of participants were collected by face-to-face interviews, including demographics, sexual behaviors, mental health, HIV knowledge, self-stigma, and internalized homophobia. Baseline CD4 at first presentation was obtained from the national integrated AIDS prevention and control information system. Logistic regression analysis was applied to analyze factors associated with LP (defined as a baseline CD4 count < 350 cells/µL). A total of 285 HIV-positive MSM participants were included, 56.5% were classified as late presenters (LPs). Multivariate regression analyses suggested that higher levels of internalized homophobia and self-stigma are positively associated with LP of HIV (both *p* < 0.001). Other linked factors included lower educational attainment (*p* = 0.039) and inadequate HIV-related knowledge (*p* < 0.001). Additionally, using severe anxiety as the reference group, individuals without anxiety symptoms showed an 86% lower likelihood of LP (*p* = 0.045, 95% CI: 0.02–0.96). Psychological barriers and a lack of related education are the potential barriers for LP of HIV among MSM in Chengdu, China. Interventions should focus on reducing homophobia and stigma while enhancing mental health support and HIV education to promote timely HIV testing in MSM.

## Introduction

Late presentation (LP) of HIV refers to a diagnosis when an AIDS-defining event (ADE) occurs or/and a CD4 cell count below 350 cells/ml^1^, which poses a threat to both individual health and public health systems^2^. Studies consistently indicate that LP is linked to higher mortality^3,4^ and elevated healthcare costs^5^ due to delayed initiation of antiretroviral therapy (ART) and missed opportunities for reducing HIV transmission through early intervention^6^.

Globally, particularly in Asia, LP remains a persistent and significant public health problem. Despite expanded testing and treatment, an estimated 34%–72% of newly diagnosed individuals in Asia still meet LP criteria^7^. Late diagnosis remains common among key populations such as MSM; for instance, South Korea continue to report substantial LP burdens^8^. Evidence from Malaysia further suggests that delayed testing is often linked to fear of disclosure, and low risk perception^9^.

Between 2005 and 2022, over half of newly diagnosed HIV/AIDS patients in China were presented late^10,11^. Among men who have sex with men (MSM), the average proportion of late presenters (LPs) was 56.53%, with a cumulative growth of 9.5% from 2015 to 2020^10^. Despite efforts to improve HIV education and testing services targeting MSM. They still face barriers to HIV testing and treatment, such as reluctance to disclose their sexual orientation due to the fear of shame^12^. Evidence^13^ has suggested that stigma, homophobia, and the fear of disclosing sexual behavior significantly hinder MSM from seeking timely HIV prevention and care services, leading to worse outcomes.

Internalized homophobia^14^ is an emotional experience and cognitive processing pattern formed in which individuals adopt negative attitudes or stereotypes about their sexual orientation, shaped by the predominantly heterosexual norms of society. Research^15^ has proposed that homosexuality and internalizing discrimination are prevalent in MSM regardless of HIV infection, especially in the elderly and married population. This emotional and cognitive distress can result in strained doctor-patient relationships, delayed medical care, and severe consequences for the health of MSM^16^. Self-stigma^17^ is also a severe psychosocial concern among Chinese MSM^18^. It refers to the internalization of negative beliefs, including discriminatory attitudes, stereotypes, or prejudices, about one’s characteristics. Various forms of stigma may reduce their willingness to undergo HIV or other sexually transmitted infection (STI) testing and diagnosis^10^. In addition, studies conducted in China^19^, India^20^ and the USA^21,22^ have shown a strong correlation between self-stigma and elevated HIV risk. Similar findings were also found in Internalized homophobia^23,24^. However, the associations between them and LP in MSM are still poorly understood.

Chengdu, Sichuan province, has a relatively high rate of HIV infection^25,26^ and mobility^27^, making it a key area for HIV detection and prevention. In light of this, this study was conducted to explore the factors related to LP of HIV/AIDS in Chengdu, particularly the psychosocial factors of internalized homophobia and self-stigma among MSM. Understanding these barriers and whether they relate to LP is urgent for designing effective interventions to promote early diagnosis and improve health among MSM^28^.

## Methods

### Study design and population

This facility-based cross-sectional study was performed at the Nuclear Industry 416 Hospital (The 2nd Affiliated Hospital of Chengdu Medical College), one of the largest HIV treatment centers in Chengdu. From 2009 to 2023, a total of 1,082 MSM living with HIV received care at this hospital. Considering the funding constraints and feasibility, we used a pre-designed simple random sampling method to represent the entire HIV/AIDS-diagnosed MSM patient population in the hospital. The sample size was estimated using the following equation:

$$\:\mathrm{n}={\left(\frac{{\mathrm{z}}_{1-{\upalpha\:}/2}}{{\updelta\:}}\right)}^{2}\:\mathrm{P}(1-\mathrm{P})$$

The parameter n represents the required sample size for the study. Where *P* (prevalence of LP) was taken as 28% which is based on a prior study conducted in China^29^. We took α as 0.05, $\:{\mathrm{z}}_{1-{\upalpha\:}/2}$ as 1.96, and 5% of $\:{\updelta\:}$ as an allowable error, the calculated sample size was 307. The inclusion criteria were: (1) participants > 18 years of age or older; (2) had ever engaged in same-sex sexual behavior; (3) having no cognitive impairments. Patients with incomplete data, such as missing CD4 test results or other required information were excluded. After excluding participants for refusal to participate or incomplete information (*n* = 22), a total of 285 patients were included. This research strictly adhered to the principles of the “WMA Declaration of Helsinki—Ethical Principles for Medical Research Involving Human Participants” and relevant Chinese laws and regulations. All participants provided voluntary informed consent after being fully informed of the research’s purpose and procedures. All personal information was anonymized throughout the research process. The research protocol was approved by the Ethics Committee of Nuclear Industry 416 Hospital (registration number: R-5M1-23-028718).

### Data collection and definition

Medical records of HIV-positive MSM between January 2009 and December 2023 were reviewed. The total number of new diagnoses during this period was summarized and categorized into NLPs and LPs. Among them, selected participants were surveyed by trained interviewers and completed a questionnaire face-to-face. Demographic data (age, education, marital status, occupation, income level, etc.), HIV-related knowledge, depression, anxiety, internalized homophobia and self-stigma were collected. Baseline CD4 is obtained from the National Integrated AIDS Prevention and Control Information System. The criteria for defining LP of HIV/AIDS is a baseline CD4 cell count of less than 350 cells/µL, an ADE reported or progression to AIDS within one year.

Knowledge about HIV/AIDS was assessed using eight questions from the HIV/AIDS core knowledge and questionnaire for HIV education and awareness, published by the China Center for Disease Control and Prevention (China CDC) in 2016^11^. Responses were scored as follows: 1 for correct answers, and 0 for both incorrect and ‘don’t know’ responses, with a maximum possible score of 8. A total score of 6 or higher was considered to have adequate HIV-related knowledge, otherwise defined as inadequate.

The Patient Health Questionnaire-9 (PHQ-9)^30^ was applied to evaluate symptoms of depression, with the full version consisting of nine items and a total score range from 0 to 27. PHQ-9 scores of 5–9, 10–14, 15–19, and 20–27 indicate mild, moderate, moderately severe, and severe depressive symptoms, respectively. The Generalized Anxiety Disorder-7 (GAD-7)^31^ was used to evaluate symptoms of anxiety. The scale includes seven items, with responses ranging from 0 (not at all) to 3 (almost every day). Scores of 5–9, 10–14, and 15–21 representing mild, moderate, and severe anxiety symptoms, respectively.

Internalized homophobia was assessed using the Short Internalized Homonegativity Scale (SIHS)^11^, a seven-item scale that evaluates the negative emotions participants may have due to their sexual orientation. Each item is rated on a scale from 1 (strongly disagree) to 7 (strongly agree), with item 5 being reverse scored. The scale comprises three dimensions of self-stigma. The scale comprises three dimensions of self-stigma including personal comfort with a gay identity, social comfort with gay men and public identification as gay. The mean score serves as the measurement indicator, with lower scores indicating more serious internalized homophobia.

Self-stigma was assessed through the Self-Stigma Scale-Short Form (SSS-S)^32,33^, an established tool for assessing the internalized stigma associated with being homosexual in China. A 4-point Likert scale was applied to rate the level of agreement with each question, ranging from 1 (strongly disagree) to 4 (strongly agree). The scale comprises nine items that assess the affective, behavioral, and cognitive dimensions of self-stigma. A higher score indicates stronger self-stigma.

### Statistical analysis

Characteristics of participants were summarized as frequencies or percentages for categorical variables, and between-group comparisons were performed using the chi-square test; Fisher’s exact test was used when the expected cell count was < 5. Continuous quantitative variables with a normal distribution were summarized as means ± standard deviations (SDs); differences between two groups were assessed using the t test, and differences among multiple groups were examined using analysis of variance (ANOVA). Continuous quantitative variables with a skewed distribution were summarized as medians and interquartile ranges (IQRs); differences between two groups were assessed using the Mann–Whitney U test, and comparisons among multiple groups were conducted using the Kruskal–Wallis test. Univariate and multivariate analyses were performed to calculate odds ratios (OR) using logistic regression. In the multivariate analysis, variables with *p* < 0.10 in univariable analyses and variables that may related to LP were included to further explore the associated factors. Both crude and adjusted odds ratios (aOR), 95% confidence interval (CI), and the corresponding *p*-values were reported. A two-tailed *p*-value below 0.05 is considered significant. Data were analyzed in R (version 4.3.1; R Core Team, 2023; R Foundation for Statistical Computing, Vienna, Austria).

## Results

The characteristics of the study population were summarized in (Table 1). Overall, the mean age of the participants was 33.98 years (SD = 8.69), and a large proportion (70.9%) had attained an education of associate or bachelor’s degree. Most participants (84.9%) were unmarried, and 13.1% reported being married or divorced. Of all participants, more than one-half of the participants (61.4%) were employed in white-collar jobs, and 72.3% reported a monthly income below 10,000 yuan. Additionally, 213 (74.7%) of the patients had adequate HIV-related knowledge. When comparing the two groups, 56.5% of the participants were classified as LPs, with a median CD4 counts of 222 cells/µL, and 43.5% were classified as NLPs, with a median CD4 counts of 450 cells/µL. While patients with low education (high school or below) often presented late (72.9%, *p* = 0.014). Moreover, the proportion of MSM without adequate knowledge was significantly higher among LPs (38.5%) compared to NLPs (8.1%) *(p* < 0.001).In our studies, 50.9% of the participants exhibited symptoms of depression (PHQ > 9), and 43.5% showed signs of anxiety (GAD > 7). More anxious symptoms were reported in LPs, especially the moderate and severe anxiety symptoms (17.4% vs. 4.8%, *p* < 0.001).

The mean score for the SIHS and SSS-S was 4.04 (SD = 1.51) and 2.22 (SD = 0.87), respectively, with LP being more common among MSM who had higher levels of internalized homophobia and self-stigma (both *p* < 0.001). “Social situations with gay men make me feel uncomfortable” on the SIHS and “I fear that others would know that I am an MSM” on the SSS-S were the most concerning items for participants. Tables 2 and 3 summarized the details of SIHS and SSS-S.

Multicollinearity was assessed using variance inflation factors (VIFs) and tolerance. All predictors had VIFs < 5 and tolerance values > 0.2, indicating no evidence of significant multicollinearity. Table 4 summarizes the factors associated with LP of HIV. Univariate and multivariate analyses consistently identified education level, HIV-related knowledge, anxiety symptoms, internalized homophobia, and self-stigma as associated factors for LP. In the multivariate analysis, individuals with an education of high school or below were more likely to experience LP compared to those who had a master’s degree or higher [ *p* = 0.039, (aOR: 4.14, 95% CI: 1.08–15.9)]. A lack of HIV-related knowledge was significantly linked to risk of LP [ *p* < 0.001, (aOR: 7.19, 95% CI: 3.20-16.15)]. Participants without anxiety symptoms were less likely to present late compared to those with severe symptoms [ *p* = 0.045, (aOR: 0.14, 95% CI: 0.02–0.96)], but we found no association between depression and LP. Lower scores on the SIHS were identified as a risk factor for LP [ *p* < 0.001, (aOR: 0.32, 95% CI: 0.20–0.52)], while lower scores on the SSS-S were associated with a decreased risk of LP. [ *p* < 0.001, (aOR: 3.46, 95% CI: 1.86–6.44)].

**Table 1:** Sociodemographic and psychological characteristics of the MSM overall, non-late presenters and late presenters.

Characteristics	Total	NLPs	LPs	*p*-value (NLPs vs. LPs)
	*n* (%)	*n* (%)	*n* (%)	
All	285 (100%)	124 (43.5%)	161 (56.5%)	
Age				0.601
18–29	98 (34.4%)	45 (36.3%)	53 (32.9%)	
30–39	124 (43.5%)	55 (44.4%)	69 (42.9%)	
40 and above	63 (22.1%)	24 (19.4%)	39 (24.2%)	
Education level				**0.014**
High school or below	59 (20.7%)	16 (12.9%)	43 (26.7%)*	
Associate or bachelor’s degree	202 (70.9%)	94 (75.8%)	108 (67.1%)	
Master’s degree or above	24 (8.4%)	14 (11.3%)	10 (6.2%)	
Marital status				0.135
Unmarried	248 (84.9%)	109 (88.7%)	133 (85.7%)	
Married	18 (6.3%)	4 (3.2%)	14 (8.7%)	
Divorced	19 (6.8%)	10 (8.1%)	9 (5.6%)	
Occupation				0.239
White collar	175 (61.4%)	81 (65.3%)	94 (58.4%)	
Blue collar	110 (38.6%)	43 (34.7%)	67 (41.6%)	
Income level				
No income	19 (6.7%)	6 (4.8%)	13 (8.1%)	0.072
Less than 2000 yuan	16 (5.6%)	7 (5.6%)	9 (5.6%)	
2000–5000 yuan	57 (20%)	24 (19.4%)	33 (20.5%)	
5000–10,000 yuan	114 (40%)	61 (49.2%)	53 (32.9%)	
10,000–20,000 yuan	65 (22.8%)	23 (18.5%)	42 (26.1%)	
More than 20,000 yuan	14 (4.9%)	3 (2.4%)	11 (6.8%)	
HIV-related knowledge				**< 0.001**
Adequate	72 (25.3%)	10 (8.1%)	62 (38.5%)*	
Inadequate	213 (74.7%)	114 (91.9%)	99 (61.5%)*	
Depression symptoms				0.716
None	140 (49.1%)	60 (31.5%)	80 (27.3%)	
Mild	83 (29.1%)	39 (48.4%)	44 (49.7%)	
Moderate	39 (13.7%)	18 (14.5%)	21 (13%)	
Moderately severe	16 (5.6%)	5 (4.0%)	11 (6.8%)	
Severe	7 (2.5%)	2 (1.6%)	5 (3.1%)	
Anxiety symptoms				**0.013**
None	161 (56.5%)	78 (62.9%)	83 (51.6%)	
Mild	90 (31.6%)	40 (32.3%)	50 (31.1%)	
Moderate	21 (7.4%)	4 (3.2%)	17 (10.6%)*	
Severe	13 (4.6%)	2 (1.6%)	11 (6.8%)*	
CD4 count, median (IQR)	324 (207–439)	450 (399–559)*	222 (139–291)*	**< 0.001**
SIHS, means ± SD	4.04 ± 1.51	4.49 ± 1.36*	3.69 ± 1.45*	**< 0.001**
SSS-S, means ± SD	2.22 ± 0.87	1.90 ± 0.74*	2.47 ± 0.85*	**< 0.001**

Bold values and *superscripts indicate significant difference between groups.

LPs, late presenters; NLPs, non-late presenters; n, number of MSM; SIHS, the short internalized homonegativity scale; SSS-S, the self-stigma scale-short form.

**Table 2:** Descriptive statistics of short internalized homonegativity scale among MSM in Chengdu.

Domain and item	Mean ± SD
Personal comfort with a gay identity (PC)	
Even if I could change my sexual orientation, I wouldn’t.	4.32 ± 1.50
I feel comfortable being a homosexual man.	3.75 ± 1.56
Homosexuality is morally acceptable to me.	4.07 ± 1.77
Social comfort with gay men (SC)	
I feel comfortable in gay bars.	4.19 ± 1.47
Social situations with gay men make me feel uncomfortable.	3.61 ± 1.24
Public identification as gay (PUBID)	
I feel comfortable discussing homosexuality in a public situation.	4.09 ± 1.52
I feel comfortable being seen in public with an obviously gay person.	4.25 ± 1.51
Mean Internalized homophobia	4.04 ± 1.51

MSM: men who have sex with men; SD: standard deviation.

**Table 3:** Descriptive statistics of self-stigma scale-short form among MSM in Chengdu.

Domain and item	Mean ± SD
Affective dimension	
I fear that others would know that I am a MSM.	2.75 ± 1.09
I feel helpless because I am a MSM.	2.46 ± 0.97
I feel uncomfortable because I am a MSM.	2.48 ± 0.93
Behavioral dimension	
I dare not to make new friends lest they find out that I am a MSM.	2.00 ± 0.79
I estrange myself from others because I am a MSM.	2.34 ± 0.85
I avoid interacting with others because I am a MSM.	1.78 ± 0.79
Cognitive dimension	
The identity of being a man who has sex with men taints my life.	2.02 ± 0.77
My identity as a MSM incurs inconvenience in my daily life.	1.90 ± 0.79
My identity as a MSM is a burden to me.	2.28 ± 0.87
Mean Self-stigma	2.13 ± 0.85

MSM: men who have sex with men; SD: standard deviation.

**Table 4:** Univariate and multivariate logistic regression analysis of factors associated with HIV late presentation.

Characteristic	Univariable analysis		Multivariable analysis	
	Crude OR (95% CI)	*p*-value	aOR (95% CI)	*p*-value
Age				
18–29	0.73 (0.38–1.38)	0.328		
30–39	0.77 (0.42–1.44)	0.413		
40 and above	1.00 (ref)	-		
Education level				
High school or below	3.76 (1.39–10.17)	**0.009**	4.14 (1.08–15.9)	**0.039**
Associate or bachelor’s degree	1.61 (0.68–3.79)	0.277	1.75 (0.57–5.44)	0.251
Master’s degree or above	1.00 (ref)	-	1.00 (ref)	
Marital status				
Unmarried	0.26 (0.06–1.08)	0.063		
Married	0.36 (0.12–1.12)	0.078		
Divorced	1.00 (ref)	-		
Occupation				
White collar	0.75 (0.46–1.21)	0.234		
Blue collar	1.00 (ref)	-		
Income level				
No income	1.00 (ref)	-		
Less than 2000 yuan	0.59 (0.15–2.37)	0.459		
2000–5000 yuan	0.64 (0.21–1.91)	0.418		
5000–10,000 yuan	0.40 (0.14–1.13)	0.084		
10,000–20,000 yuan	0.84 (0.28–2.51)	0.759		
More than 20,000 yuan	1.69 (0.34–8.40)	0.520		
HIV-related knowledge				
Adequate	1.00 (ref)	-	1.00 (ref)	-
Inadequate	7.14 (3.48–14.67)	**< 0.001**	7.19 (3.20-16.15)	**< 0.001**
Anxiety symptoms				
None	0.19 (0.04–0.90)	**0.036**	0.14 (0.02–0.96)	**0.045**
Mild	0.23 (0.05–1.09)	0.063	0.25 (0.04–1.70)	0.162
Moderate	0.77 (0.12–4.96)	0.786	0.63 (0.07–5.93)	0.685
Severe	1.00 (ref)	-	1.00 (ref)	-
SIHS	0.29 (0.20–0.42)	**< 0.001**	0.32 (0.20–0.52)	**<0.001**
**SSS-S**	4.68 (2.95–7.44)	**< 0.001**	3.46 (1.86–6.44)	**<0.001**

Bold values indicate statistical significance.

aOR, adjusted odds ratio; CI, confidence interval; OR, odds ratio; SIHS, the short internalized homonegativity scale; SSS-S, the self-stigma scale-short form.

## Discussion

This study examined the prevalence of LP of HIV among MSM in Chengdu, China. The results showed a high proportion of LP cases, which is similar to recent studies^34,35^ from China but higher than the average risk reported in the general population^36^. Our findings suggested that educational level, HIV-related knowledge, anxiety, internalized homophobia, and self-stigma were associated with LP of HIV among MSM in Chengdu. Notably, we did not find a significant association between LP and socio-demographic factors including age, marital status, or income.

Consistent with studies from Spain^3^ and Norway^37^, our study found that low levels of education were associated with a higher likelihood of LP among MSM. One of the main factors associated with late presentation in this study was limited HIV-related knowledge. This finding aligns with a study conducted in Switzerland^38^, which reported insufficient knowledge about HIV transmission and symptoms as the primary factor linked with delayed testing. Our findings link poor mental health to LP of HIV, this has been the case in a previous study^39^. The anxiety rate among MSM in our study was 43.5%, which is higher than the anxiety rate of 21.8% in the general Chinese population^40^. This study found that higher levels of internalized homophobia and self-stigma are independently associated with increased odds of LP. Evidence^24,41,42^ has promoted that internalized homophobia and stigma significantly reduce testing behaviors and adherence to treatment among MSM. Other research from Ethiopia^43^ also found that fear of stigma and discrimination was more common among individuals who were late for HIV diagnosis.

This result may explained by the fact that lower educational attainment often limits health literacy, restricts access to accurate HIV-related information, and reduces engagement with preventive services. Previous research^44^ has reported that individuals with lower health literacy are more likely to delay HIV testing. Conversely, higher education levels tend to foster greater engagement with healthcare services, leading to earlier HIV diagnoses^45^. Without adequate knowledge, patients may fail to recognize early symptoms, leading to reduced risk perception and delays in testing^37,46^. These findings underline the importance of targeted interventions aimed at addressing the potential knowledge gap, particularly in high-risk populations such as MSM, and it has been proven effective in the USA^47^.

Mental health is highly prevalent among MSM in China^48^. Depression and anxiety have been identified as key risk factors for Unprotected Anal Intercourse (UAI) and poor HIV care^49–51^, which increases the risk of HIV infection and LP in MSM. Considering that HIV-infected individuals with poor mental health related to have higher mortality rates^52^, comprehensive medical services are needed to address the psychological burden of LP.

These findings reflected their pervasive impact on healthcare access among MSM. Internalized homophobia and self-stigma create psychological barriers^53^, indirectly affect social functioning^54^ and self-esteem^55^, reduce the likelihood of seeking health-promoting behaviors^56^, and hinder MSM from accessing HIV testing services^57^. Addressing these issues through stigma-reduction interventions and fostering supportive environments are essential for improving timely HIV diagnosis. Interventions such as community-based peer support and education programs have proven effective in reducing psychological barriers and promoting earlier healthcare engagement^58^.

This lack of association may reflect the homogeneity of the management. MSM in the city are managed by regional disease control and community health service centers, ensuring high availability of HIV testing, which may mitigate the impact of demographic variables and equalize access across different groups^59,60^. Moreover, stigma associated with sexual orientation and poor mental health may have a greater impact on LP, suggesting that integrating mental health support into HIV care services may be more urgent than focusing solely on demographic predictors^61^.

This study has several disadvantages. First, the cross-sectional design limits the ability to draw causal conclusions about the relationships between variables and LP of HIV. Second, Because the social–psychological variables were measured at the time of the questionnaire survey, whereas LP reflects diagnostic status at the initial visit or confirmation, we cannot rule out the possibility that individuals diagnosed at a late stage may subsequently report higher levels of stigma or anxiety after diagnosis, rather than these factors leading to LP. Third, the study population was drawn from a single tertiary hospital in Chengdu, which may restrict the ability to generalize the findings to other regions or healthcare environments.

## Conclusions

These findings underscore the need for multifaceted interventions including enhancing awareness about HIV/AIDS, developing targeted strategies for internalized homophobia and self-stigma reduction, especially among individuals with low educational attainment. Given the high prevalent of LP among MSM, more epidemiological studies are needed to confirm these associations and further improve early diagnosis among MSM living with HIV.

## Data Availability

The dataset analyzed during the current study is not publicly available to protect the privacy of study participants given the small size of the recruited sample. However, de-identified personal data can be obtained from the corresponding author upon reasonable request.
